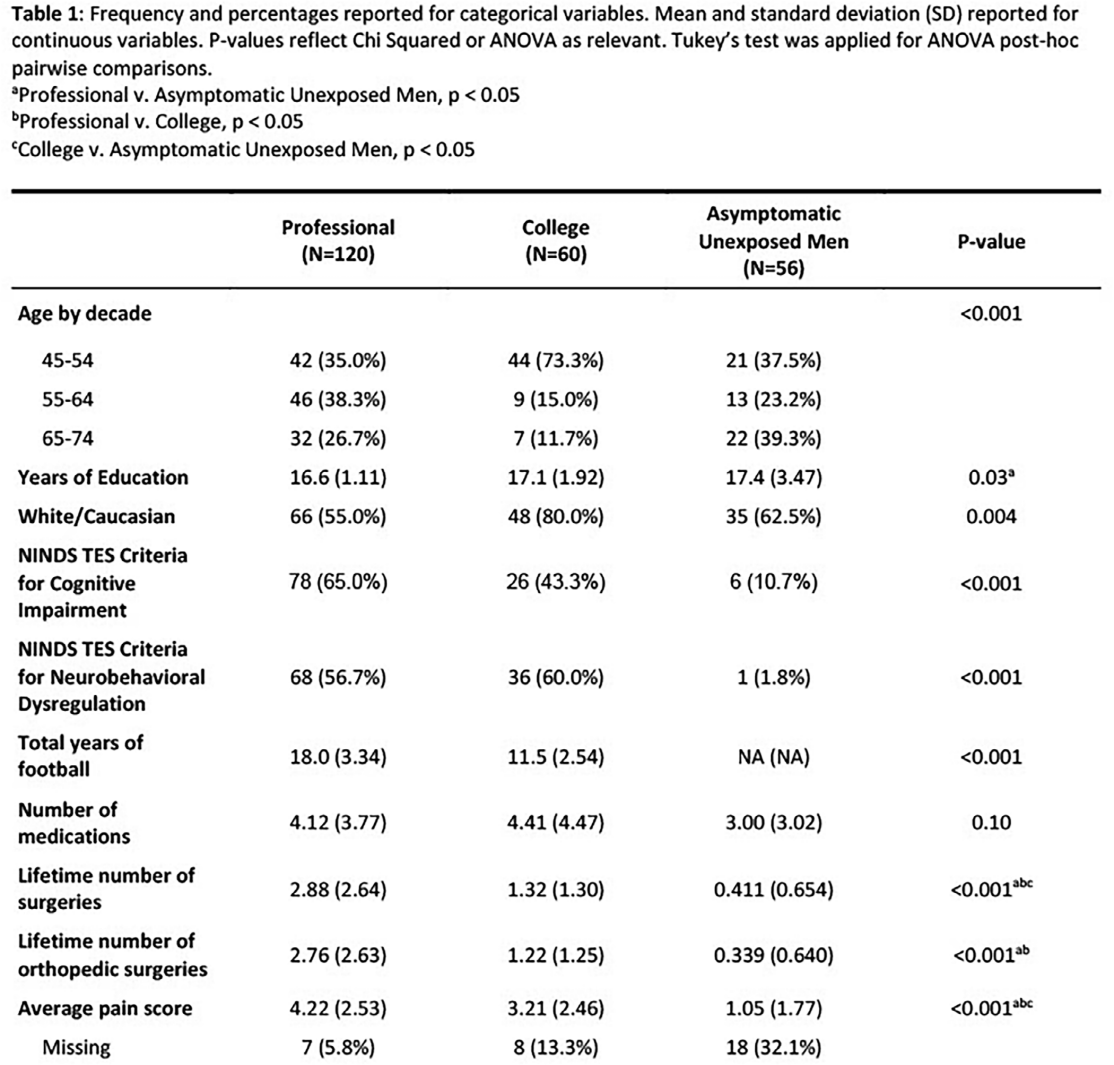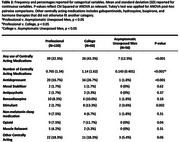# Association of Centrally Acting Medications, Chronic Pain, and Orthopedic Surgical History with Cognitive Impairment and Neurobehavioral Dysregulation in Former American Football Players

**DOI:** 10.1002/alz70861_108461

**Published:** 2025-12-23

**Authors:** Alexa Puleio, Ina Hoti, William B. Barr, Sarah J Banks, Jennifer V. Wethe, Yorghos Tripodis, Charles Adler, Laura Balcer, Charles B. Bernick, David W. Dodick, Robert C Cantu, Douglas I Katz, Jesse Mez, Joseph N. Palmisano, Brett Martin, Jeffrey L. Cummings, Eric M. Reiman, Martha E. Shenton, Robert A. Stern, Michael L Alosco, Steven Lenio, DIAGNOSE CTE Research Project

**Affiliations:** ^1^ Boston University Chobanian & Avedisian School of Medicine, Boston, MA USA; ^2^ Boston University Alzheimer’s Disease Research Center, Boston, MA USA; ^3^ NYU Langone Health, New York City, NY USA; ^4^ University of California, San Diego, La Jolla, CA USA; ^5^ Mayo Clinic, Scottsdale, AZ USA; ^6^ Boston University School of Public Health, Boston, MA USA; ^7^ Parkinson's Disease and Movement Disorders Center, Mayo Clinic, Scottsdale, AZ USA; ^8^ NYU Grossman School of Medicine, New York, NY USA; ^9^ Lou Ruvo Center for Brain Health, Cleveland Clinic, Las Vegas, NV USA; ^10^ Concussion Legacy Foundation, Boston, MA USA; ^11^ Boston University Chronic Traumatic Encephalopathy Center, Boston, MA USA; ^12^ Kirk Kerkorian School of Medicine, University of Nevada Las Vegas, Las Vegas, NV USA; ^13^ Banner Alzheimer's Institute, Phoenix, AZ USA; ^14^ Brigham and Women's Hospital, Boston, MA USA

## Abstract

**Background:**

Exposure to repetitive head impacts (RHI) is associated with developing chronic traumatic encephalopathy (CTE) neuropathology. Cognitive and behavioral symptoms have been associated with CTE neuropathology, but efforts to define the specific clinical syndrome are ongoing. This study characterizes the current use of centrally acting medications (CAMs), chronic pain, and number of orthopedic surgeries in former American football players and evaluates for associations with cognitive and behavioral symptoms.

**Methods:**

This study analyzed data from the DIAGNOSE CTE Research Project, which includes 120 former professional football players, 60 collegiate football players, and 56 asymptomatic men without history of RHI. Medical history was obtained by self‐report, and participants completed the Montreal Cognitive Assessment (MoCA), Brief Pain Inventory, Barratt Impulsiveness Scale‐11 (BIS‐11), BRIEF‐A Behavioral Regulation Index (BRI), Beck Depression Inventory‐II (BDI‐II), Beck Anxiety Index (BAI), and Brown‐Goodwin Lifetime History of Aggression adulthood score (BGLHA). Logistic regression assessed the association of CAMs, average pain score, and orthopedic surgeries on the cognitive impairment and neurobehavioral dysregulation components of the NINDS traumatic encephalopathy syndrome (TES) research criteria, in former American football players. Linear regression assessed the associations with MoCA and behavioral scales. Models were adjusted for age, education, race, and years of football played.

**Results:**

Tables 1 and 2 describe the sample and CAM burden. More orthopedic surgeries, CAMs, and higher average pain were observed in former American football players. Number of CAMs was associated with neurobehavioral dysregulation (OR = 2.18), lower MoCA (β = ‐0.47), and higher BIS‐11 (β = 2.23), BDI‐II (β = 2.01), BRI (β = 3.71), BAI (β = 2.04), and BGLHA (β = 0.68) scores. Higher average pain was associated with neurobehavioral dysregulation (OR = 1.57) and higher BIS‐11 (β = 2.20), BDI‐II (β = 1.98), BRI (β = 2.20), BAI (β = 1.84), and BGLHA (β = 0.52) scores. No associations were observed with orthopedic surgeries.

**Conclusions:**

In former American football players, the number of CAMs and higher average pain were associated with neurobehavioral dysregulation. No associations were observed with diagnosis of cognitive impairment by TES criteria, though the number of CAMs was associated with decreased MoCA.